# Function‐related Drivers of Skull Morphometric Variation and Sexual Size Dimorphism in a Subterranean Rodent, Plateau Zokor (*Eospalax baileyi*)

**DOI:** 10.1002/ece3.3986

**Published:** 2018-04-15

**Authors:** Junhu Su, Ibrahim M. Hegab, Weihong Ji, Zhibiao Nan

**Affiliations:** ^1^ State Key Laboratory of Grassland Agro‐ecosystems College of Pastoral Agriculture Science and Technology Lanzhou University Lanzhou China; ^2^ College of Grassland Science Key Laboratory of Grassland Ecosystem (Ministry of Education) Gansu Agricultural University Lanzhou China; ^3^ Gansu Agricultural University‐Massey University Research Centre for Grassland Biodiversity Gansu Agricultural University Lanzhou China; ^4^ Faculty of Veterinary Medicine Department of Hygiene, Zoonosis and Animal Behavior & Management Suez Canal University Ismailia Egypt; ^5^ Institute of Natural and Mathematical Sciences Massey University Auckland New Zealand

**Keywords:** natural selection, plateau zokor, Rensch's rule, sexual size dimorphism, subterranean rodents

## Abstract

Sexual dimorphism is prevalent in most living organisms. The difference in size between sexes of a given species is generally known as sexual size dimorphism (SSD). The magnitude of the SSD is determined by Rensch's rule where size dimorphism increases with increasing body size when the male is the larger sex and decreases with increasing average body size when the female is the larger sex. The unique underground environment that zokors (*Eospalax baileyi*) live under in the severe habitat of the Qinghai‐Tibetan Plateau (QTP) could create SSD selection pressures that may or may not be supported by Rensch's rule, making this scientific question worthy of investigation. In this study, we investigated the individual variation between sexes in body size and SSD of plateau zokors using measurements of 19 morphological traits. We also investigated the evolutionary mechanisms underlying SSD in plateau zokors. Moreover, we applied Rensch's rule to all extant zokor species. Our results showed male‐biased SSD in plateau zokors: The body‐ and head‐related measurements were greater in males than in females. Linear regression analysis between body length, body weight, and carcass weight showed significant relationships with some traits such as skull length, lower incisor length, and tympanic bulla width, which might support our prediction that males have faster growth rates than females. Further, the SSD pattern corroborated the assumption of Rensch's rule in plateau zokors but not in the other zokor species. Our findings suggest that the natural underground habitat and behavioral differences between sexes can generate selection pressures on male traits and contribute to the evolution of SSD in plateau zokors.

## 1 INTRODUCTION

Sexual dimorphism is a widespread phenomenon where males and females of the same species display different physical traits (Fairbairn & Roff, 2006; Ralls, 1977). These variations are not only constrained to their sex organs (Glucksmann, 1974), but extend further beyond to also include their size (Noonan et al., 2016; Rudoy & Ribera, 2017), behavior (Turgeon, Townsend, Dixon, Hickman, & Lee, 2016), and coloration (Cooper, Brown, & Getty, 2016). The difference in body size is a fundamental feature of sexual dimorphism (Jimenez‐Arcos, Sanabria‐Urban, & Cueva Del Castillo, 2017) and is termed sexual size dimorphism (SSD), which is usually linked to the intraspecific differences in ecology, life history, and behavior between sexes (Halámková, Schulte, & Langen, 2013; Nevo & Beiles, 2010; Šumbera, Burda, & Chitaukali, 2003). SSD is common in nature (Han & Fu, 2013) and includes different types, male‐biased and female‐biased (Wu et al., 2014). Male‐biased SSD (MBSSD) can be seen in most mammalian species and lizards, where males have larger body size than females (Hudson & Fu, 2013; Kinahan, Bennett, O'Riain, Hart, & Bateman, 2007). Conversely, female‐biased SSD (FBSSD) pattern can be seen in invertebrates, ectothermic vertebrates, and some mammalian species, where females are larger than males (Chelini & Hebets, 2017; Kilanowski & Koprowski, 2017). Despite extensive research on this topic, a critical question remains unanswered: Why do males and females manifest different body sizes, although they possess the same genes of growth and development?

Evolutionary biologists have identified major selective forces on SSD. Several hypotheses have been proposed to clarify the development of SSD. The sexual selection hypothesis (Blanckenhorn et al., 2007) predicts that larger males might compete more efficiently to gain access to mates for better reproductive success and this drives MBSSD (Stillwell, Blanckenhorn, Teder, Davidowitz, & Fox, 2010). The fecundity selection theory states that larger females would produce higher numbers of healthier offspring and/or reproduce more frequently than smaller ones, thereby driving FBSSD or “reversed” sexual size dimorphism (Pincheira‐Donoso & Hunt, 2017). An alternative, sex‐based ecological divergence (Brown, Madsen, & Shine, 2017) hypothesis states that SSD evolves to facilitate different sexes to exploit the available resources differently to reduce competition between them (Szekely, Reynolds, & Figuerola, 2000). The pioneer work of Bernhard Rensch revealed what we know as “Rensch's rule” (Rensch, 1959). The rule is, when females are larger, the relative degree of SSD decreases with body size (hypoallometry; Abouheif & Fairbairn, 1997); however, among species in which males are larger, the relative SSD increases with body size (hyperallometry; Webb & Freckleton, 2007). Statistically, the relationship between the magnitude of SSD and body size is the best clarified by means of an allometric relationship. For example, the allometric slopes in MBSSD would be less than one when female size is regressed on male size (Abouheif & Fairbairn, 1997). Although Rensch's rule was verified in different species (Halámková et al., 2013), it has been an issue of controversial debate with respect to its general validity and application to developmental processes that would result in this macroevolutionary patterns (StarostovÁ, KubiČKa, & KratochvÍL, 2010). One of the drawbacks of Rensch's rule is that it is well supported for most (but not all) cases that exhibit male‐biased or mixed SSD at the interspecific and intraspecific levels, but the patterns of allometry among taxa with FBSSD are less clear (Liao, Liu, & Merila, 2015; Webb & Freckleton, 2007).

**Figure 1:**
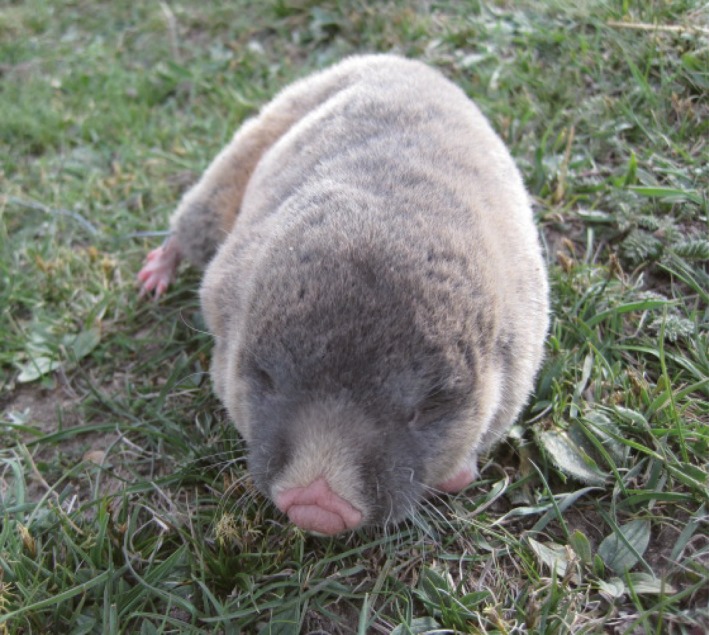
Plateau Zokor (*Eospalax baileyi*)

Plateau zokor (*Eospalax baileyi*) is a typical subterranean rodent species inhabiting the Qinghai‐Tibetan Plateau (Fig. 1) (QTP; Su, Aryal, Nan, & Ji, 2015), the highest and largest plateau in the world. Because of the high altitude, with an average elevation exceeding 4,500 m, the species inhabiting the QTP experience both low oxygen tension (hypoxia) (Shao et al., 2015; Zhao et al., 2016) and harsh environmental conditions (Xu et al., 2016), with permafrost covering about half of its total area (Yi, Wang, Qin, Xiang, & Ding, 2014). Plateau zokors spend the greater part of their life in underground tunnels except for very few instances for aboveground foraging (Zhou & Dou, 1990). Like other subterranean rodents, plateau zokors show morphological, physiological, and behavioral adjustments for the various underground activities (Wang et al., 2012). For example, changing burrowing strategy is a solution to avoid unnecessary digging activities and increase net energy yield per given section of burrow such as construction of a narrow burrow or decrease the burrow length (White, 2005). Also, reduction in certain organs such as eyes and external ears coupled with development of other organs as incisors and forelimbs may optimize the efficiency of the digging process (Nevo, 1979). However, some subterranean rodent species exhibit SSD (such as Cape dune mole‐rats, *Bathyergus suillus* and Namaqua mole‐rats, *Bathyergus janetta*) and others do not (such as Cape mole‐rats, *Georychus capensis*; Bennett & Faulkes, 2000). This controversy had been thoroughly explained by (Martínez & Bidau, 2016) that the underground environment may possess some degree of constraints over the development of SSD in subterranean rodents. However, in the same study and a previous one (Bidau Claudio & Medina Alonso, 2013), tuco‐tucos (*Ctenomys perrensi*) showed clear SSD, but Rensch's rule is not verified which affirmed that both SSD and the allometry with Rensch's rule might be mutually exclusive. Similarly, the pattern of digging and home‐range sizes significantly differ between male and female zokors, males tend to have larger home ranges and use spectacular way of digging with males start to dig longitudinal tunnels with two‐branched ends to intercept female burrows and increase the chances to trap the females while females have smaller home ranges with circular pattern of burrowing (Hegab et al., 2018; Zhang, 2007). Therefore, in this study we firstly are trying to assess whether the direction of SSD in plateau zokors is MBSSD or FBSSD by determining the correlation between different morphological and anatomical features. Moreover, we tested the allometric relationship predicted by Rensch's rule using seven zokor species to estimate the degree of SSD between males and females in addition to plateau zokors. The findings of this study might provide more data regarding the evolution of SSD in subterranean rodents.

## 2 MATERIALS AND METHODS

### 2.1 Location of the study

The study site is located in Gahai Town, Luqu County, Gannan Tibetan Autonomous Prefecture in the southwestern part of Gansu Province (101°35ʹ36ʺ‐102°58ʹ15ʺE, 33°58ʹ21ʺ‐34°48ʹ48ʺN), situated at the junction between the eastern edge of the QTP, Gansu, Qinghai, and Sichuan Provinces. This region extends 126 km from east to west and 93 km from north to south, with a total area of 5,298 km^2^. The annual average temperature is 2.3°C, and the annual average precipitation is 633–782 mm.

### 2.2 Animals and body measurements

Fourteen populations of wild‐caught plateau zokors were trapped and killed under the yearly control and management practice of local rodent control and prevention authorities (Su, Peng, Nan, Ji, & Cai, 2018). The following information and measurements were recorded for each individual zokor captured: the capture location; body weight (BW); carcass weight (CW, measured to ±0.01 g using an electronic balance; Shanghai Hochoice Electric Appliance Co., Ltd., Shanghai, China), which included body length (BL), tail length (TL), hindfoot length (HFL), forefoot length (FFL), skull full length (SL), skull basal length (SBL), upper incisor length (DL), greatest mastoid width (OW), mandibular length (JL), lower incisor length (UDL), orbital width (OB), nasal bone length (NL), and median palatal length (MPL, measured using a digital caliper; Guilin Digital Measurement Co., Ltd., Guilin, China, and a ruler with accuracy of 0.01 mm; Table 1; Xia, Yang, Ma, Feng, & Zhou, 2006). Bias was minimized by ensuring that the same person measured all parameters. Animals were sexed, and, within each sex, individuals were classified into five age‐groups (Su et al., 2018) according to CW: I (M: 144–210) (F: 106–148); II (M: 211–276) (F: 149–190); III (M: 277–342) (F: 191–232); IV (M: 343–408) (F: 233–274); and V (M: ≥409) (F: ≥275). The last group (V) represented very old or senile animals. The CW was also measured to omit possible confounding parameters such as visceral fat and gravid uterus in pregnant females. Testicular size for males and fetus number in killed pregnant females were also recorded.

**Table 1:** List of abbreviations for different morphological traits in plateau zokors

Abbreviations	Trait	Abbreviations	Trait
BL	Body length	OB	Orbital width
BW	Body weight	OW	Greatest mastoid width
CW	Carcass weight	SL	Skull full length
DL	Length of upper incisors	SBL	Skull base length
FFL	Forefoot length	TBL	Tympanic bulla length
HFL	Hindfoot length	TBW	Tympanic bulla width
JL	Mandibular length	TL	Tail length
ML	Diastema length	UDL	Length of lower incisors
MPL	Median palatal length	ZW	Zygomatic width
NL	Nasal bone length		

To test Rensch's rule, we used the traditional mathematical approach by constructing a linear regression model with log body length of males (*y*‐axis) and females (*x*‐axis) of plateau zokors and seven other zokor species of the genus *Myospalax*, subfamily Mysopalacinae [*M. psilurus* (82 females, 73 males) and *M. aspalax* (65 females, 57 males)], and of the *Eospalax* genus [*E. fontanierii* (27 females, 34 males), *E. cansus* (98 females, 89 males), *E. rufescens* (14 females, 13 males), *E. smithi* (14 females, 13 males), and *E. rothschildi* (39 females, 23 males)] **(**Guo et al., 2000; Luo et al., 2000; Lu, Zhang, & Zhou, 2013 **)**. Alternatively, we used phylogenetic reduced major axis (pRMA) regression with the aid of the “phytools” package in the R.3.0.2 platform. pRMA regressions in the form of log_10_ (male body mass) on log_10_ (female body mass) were executed to evaluate the slope which is an estimator of the scaling of SSD with body mass. The previous approach was adopted from Martínez and Bidau (2016). We also used a Model II regression method (RMA) in plateau zokors as ordinary least‐squares (OLS) regression is inadequate for this type of analysis in Ref. (Bohonak & Linde, 2004). Plateau zokors were trapped and killed by the local rodent control authorities of Gannan, Gansu Province. Experimental procedures were approved by the animal ethics committee of Gansu Agricultural University as well as permission was obtained from the local authorities.

### 2.3 Statistical analysis

SPSS 22.0 (Armonk, NY: IBM Corp.) software and Excel 2016 (Microsoft)^®^ software were used for data processing and statistical analysis. A *t* test was conducted on the morphological traits between males and females. Pearson's correlation coefficient was used to measure the degree of correlation between body length in both sexes and other morphological traits. Two‐way analysis of variance was conducted using age and sex as fixed variables and body and tail length as dependent variables. Linear regression models were generated based on correlations between various anatomical measurements and BW, CW, and BL in both sexes. The level of significance at which the null hypothesis was rejected was α = 0.05. Data values are represented as means ± *SE*.

## 3 RESULTS

### 3.1 Morphological and anatomical parameters of sexual dimorphism in plateau zokors

Our results showed that SSD was consistently male‐biased in plateau zokors, with males being slightly larger than females. The results showed that the BL, SL, SBL, DL, ML, TBL, TBW, NL, ZW, OW, MPL, UDL, JL, TL, FFL HFL, BW, and CW were higher in males than in females (please refer to Table 1 for abbreviations). Although *t* test did not show any significant difference between the two sexes (*t *=* *0.804, *p *=* *.211), results from Table 2 showed that the BL in males was higher than in females, and the male‐to‐female BL ratio was 1.100, calculated by dividing the means of BL of both sexes. The SL, SBL, DL, ML, TBL, TBW, NL, OW, MPL, UDL, JL, ZW, TL, FFL, and HFL were significantly correlated to BL in both sexes (Table 2). Both the sexes did not show any significant correlation between OB and BL.

**Table 2:** Sexual dimorphism of morphological and anatomical traits in plateau zokors including Mean ± *SE*, ranges, and correlation coefficients of the different studied traits in each sex with body length

Morphological trait/unit	Female			Male			*p* value
	*n*	Mean ± *SE*	Correlation Coefficient with BL (*p*)	*n*	Mean ± *SE*	Correlation coefficient with BL (*p*)	
BL/cm	260	24.08 ± 0.09 (19.8–26.9)	–	211	26.44 ± 0.13 (21.50–33.20)	–	.000
SL/mm	163	43.12 ± 0.02 (16.90–48.40)	0.220^a^ (.050)	160	46.33 ± 0.16 (41.20–51.80)	0.327^b^ (.000)	.000
SBL/mm	165	41.79 ± 0.14 (32.10–47.20)	0.366^b^ (.000)	158	45.10 ± 0.18 (39.70–50.40)	0.271^b^ (.001)	.000
DL/mm	192	9.15 ± 0.04 (8.70–10.60)	0.323^b^ (.000)	178	10.06 ± 0.03 (9.10–11.30)	0.377^b^ (.000)	.000
ML/mm	188	14.63 ± 0.05 (12.60–1.7.10)	0.420^b^ (.000)	177	15.72 ± 0.07 (13.50–19.20)	0.310^b^ (.000)	.000
TBL/mm	184	9.20 ± 0.03 (7.80–10.40)	0.364^b^ (.000)	169	9.55 ± 0.03 (8.10–10.40)	0.187^a^ (.015)	.000
TBW/mm	184	7.20 ± 0.03 (6.40–8.50)	0.166^a^ (.025)	169	7.46 ± 0.03 (6.50–8.50)	0.169^a^ (.028)	.002
OB/mm	183	8.14 ± 0.03 (7.20–9.20)	‐0.029 (.695)	171	8.27 ± 0.03 (7.10–9.10)	0.128 (.097)	.289
NL/mm	175	15.60 ± 0.07 (13.30–18.60)	0.384^b^ (.000)	172	17.01 ± 0.11 (7.80–21.40)	0.329^b^(.000)	.000
ZW/mm	177	27.45 ± 0.11 (24.50–32.40)	0.420^b^ (.000)	167	30.23 ± 0.16 (24.90–34.90)	0.251^b^ (.003)	.000
OW/mm	188	18.81 ± 0.06 (16.90–22.00)	0.351^b^ (.000)	177	20.01 ± 0.10 (10.10–28.20)	0.292^b^ (0.000)	.000
MPL/mm	201	9.93 ± 0.02 (9.00–11.30)	0.417^b^ (.000)	183	10.26 ± 0.03 (9.30–11.50)	0.304^b^(.000)	.000
UDL/mm	198	28.07 ± 0.10 (17.40–31.20)	0.267^b^ (.000)	181	29.82 ± 0.11 (26.00–34.70)	0.287^b^ (.000)	.000
JL/mm	133	29.66 ± 0.14 (26.80–35.40)	0.315^b^ (.000)	134	32.9 ± 0.20 (26.40–39.30)	0.346^b^ (.000)	.000
TL/mm	259	4.35 ± 0.03 (3.2–5.8)	0.291^b^ (.000)	203	4.72 ± 0.14 (3.5–7.3)	0.459^b^ (.000)	.000
FFL/mm	221	3.1 ± 0.01 (2.1–3.6)	0.166^a^ (.000)	189	3.2 ± 0.01 (2.5–4.0)	0.472^b^ (.000)	.000
HFL/mm	221	3.3 ± 0.01 (2.8–3.9)	0.206^b^ (.000)	189	3.6 ± 0.02 (3.0–4.6)	0.488^b^(.000)	.000

a Significant at *p *≤* *.05.

b Significant at *p *≤* *.01.

Independent *t* test showed no significant difference in OB between the two sexes, whereas SL, SBL, DL, ML, TBL, TBW, NL, OW, MPL, UDL, JL, ZW, TL, FFL, and HFL were significantly different between sexes (*p *<* *.05; Table 2). An independent *t* test (Table 3) showed that BW and CW of male plateau zokors were significantly higher than those of females (*t *=* *19.995, *p *=* *.032; and *t *=* *18.850, *p *<* *.0001, respectively).

**Table 3:** Carcass and body weights in different sexes of plateau zokors

Morphological trait	Sex	*n*	Range	Mean ± *SE*	*p* value
CW (g)	♀	260	106.80–314.5	194.43 ± 1.99	*t *= 19.995
	♂	209	144.70–473.00	294.46 ± 4.80	*p* = .032
BW (g)	♀	260	158.5–454.3	274.10 ± 2.73	*t* = 18.850
	♂	210	207.2–594.4	386.56 ± 5.72	*p* = .000

### 3.2 Ontogenetic tendency of SSD in plateau zokors

The carcasses of plateau zokors were collected and classified into five age‐groups: I, II, III, IV, and V to investigate SSD in males and females under the same age. The above‐mentioned parameters were measured for both sexes in each age‐group. In group I, independent sample *t* test showed that males had statistically higher means for all the measured parameters (*p *<* *.05, Table S1) than females, except for DL, TBW, OB, ZW, FFL, and UDL. In contrast, in groups II and III, all measured parameters were statistically higher in males than in females, except OB, which showed a nonsignificant difference between sexes. In group IV, TBL and OB did not show any significant SSD, whereas all other parameters were significantly higher in males than in females. No statistically significant difference was noted between sexes in group V with regard to DL, UDL, FFL, and OB, but other parameters were statistically higher in males than in females.

Analysis of age and sex fixed variables using two‐way ANOVA revealed that both age (*F* = 38.11, *p *=* *.00) and sex (*F* = 86.55, *p *=* *.00) had significant effects on BW in plateau zokors, whereas the age × sex interaction did not have any significant effect (*F* = 2.06, *p *=* *.09). The BLs of males were significant higher than those of females (26.15 ± 0.12 and 24.16 ± 0.18, respectively), and Duncan's post hoc test showed that the highest BL was found in groups IV and V (26.70 ± 0.34 and 26.37 ± 0.43, respectively) compared to other age‐groups. Moreover, sex (*F* = 17.79, *p *=* *.00) had a significant effect on TL, where males had longer tails than females (4.95 ± 0.05 and 4.56 ± 0.08, respectively), whereas neither age (*F* = 1.37, *p *=* *.24) nor age × sex interaction (*F* = 1.41, *p *=* *.23) had significant effects on TL.

### 3.3 Multiple linear regression analyses between different traits

**Figure 2:**
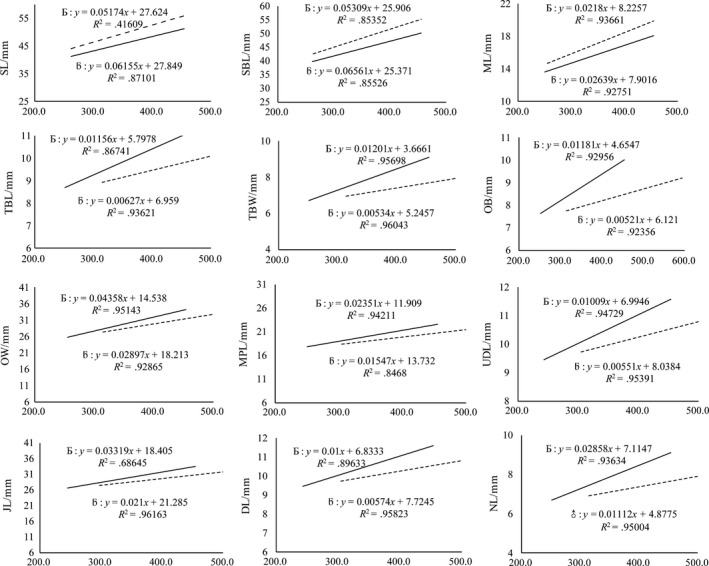
Linear regression between mean male and female body weight (g) with different traits

**Figure 3:**
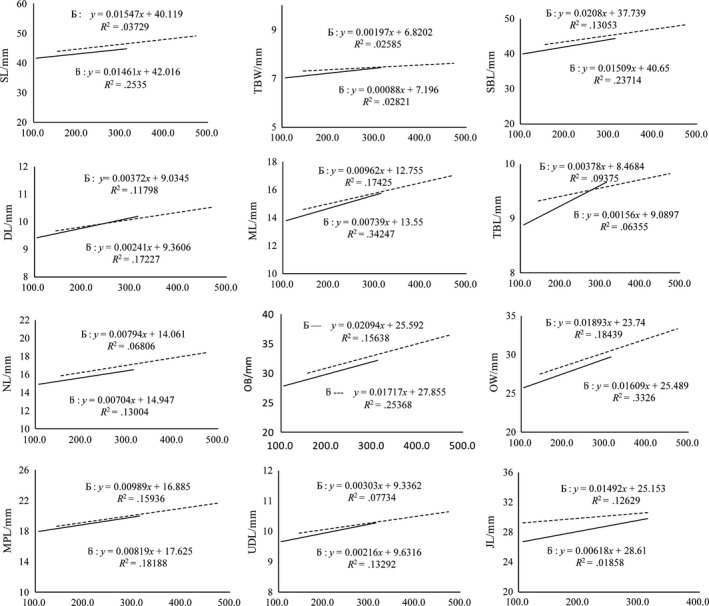
Linear regression between mean male and female carcass weight (g) with different traits

**Figure 4:**
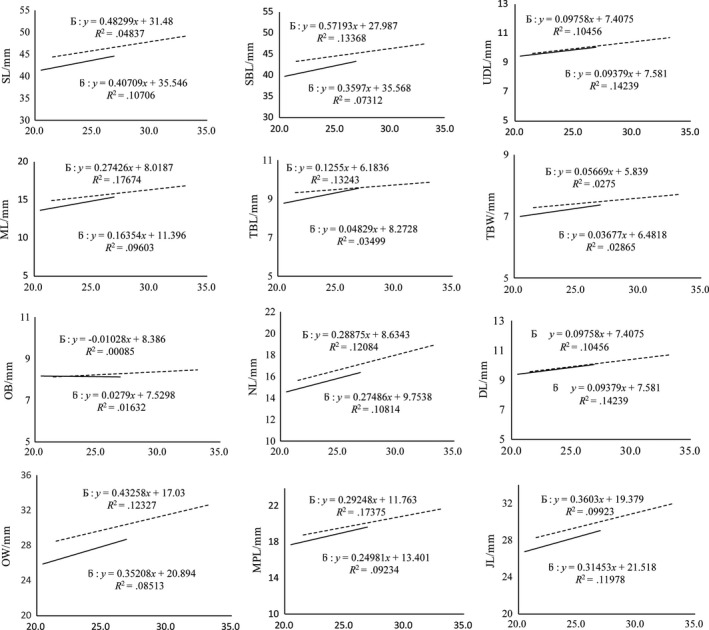
Linear regression between mean male and female body length (cm) with different traits

Linear regression models were developed to determine whether a relationship existed between SL, SBL, DL, ML, TBL, TBW, OB, NL, OW, MPL, UDL, JL, and ZW and BW, CW, and BL of both sexes (Figures 1, 2, 3, and 4, respectively).

#### 3.3.1 Linear regression and correlation between various traits and BW in plateau zokors

The degree of correlation between BW of the female zokors and ML, OB, OW, and MPL was found to be greater than that in males. In contrast, the degree of correlation between BW of the male zokors and SL, SBL, DL, TBL, TBW, NL, UDL, and JL was greater than that in females (Figure 2).

#### 3.3.2 Linear regression and correlation between various traits and CW in plateau zokors

The degree of correlation between CW of the female zokors and TBL and JL was found to be greater than that in males. In contrast, the degree of correlation between CW of the male zokors and SL, SBL, DL, OB, ML, UDL, TBW, NL, OW, and MPL was greater than that in females (Figure 3).

#### 3.3.3 Linear regression and correlation between various traits and BL in plateau zokors

The degree of correlation between BL of female zokors and SBL, ML, TBL, NL, OW, and MPL was greater than that in males. Further, the degree of correlation between BL of male zokors and SL, DL, TBW, OB, UDL, and JL was greater than that in females (Figure 4).

### 3.4 Testing Rensch's rule in plateau zokors and other zokor populations

**Figure 5:**
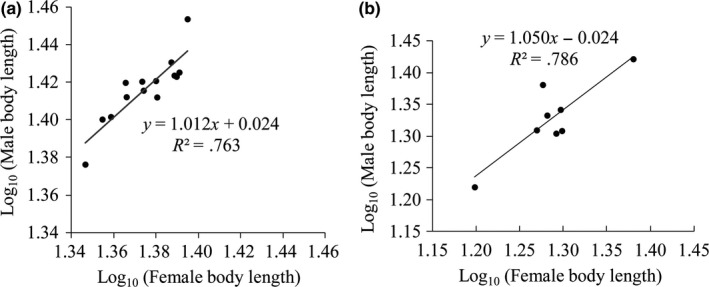
Linear regression between log (male body size) on log (female body size) in plateau zokors (a) and that of plateau zokors with eight other zokor species (b)

The first traditional approach showed that the variation pattern in SSD in plateau zokors and among different zokor populations was consistent with Rensch's rule, with male size being more variable than female size. The slope of the major axis regression of log_10_ (male body length) on log_10_ (female body length) in plateau zokors was significantly different from 1 (*R* ^2^ = .7628, slope = 1.0301, *p *=* *.00; Figure 5a), and in other zokor populations, it was 1 (*R* ^2^ = .786, slope =  1.0504, *p *=* *.001; Figure 5b). However, the scaling of SSD when analyzed by pRMA regression yielded contradictory results. The model residuals do not show phylogenetic signal (λ = 6.6e‐05), and βpRMA was not significantly different from 1.0 (isometry), signaling the nonconsistence with Rensch's rule (β = 1.20, α = −0.22, *r* ^2^ = .72, *t* = 0.85, *df *= 6.42, *p *=* *.43) on the contrary to the previous results in the zokors species. The negative value of the intercept indicates that, despite the few exceptions, the general tendency for SSD in zokors is to be male‐biased. The RMA analysis for plateau zokors signals concordance with Rensch's rule (*R* ^2^ = .76, slope = 1.159, 95% confidence interval = 0.8039–1.513).

### 3.5 Litter size and testicular size in female and male plateau zokors

**Figure 6:**
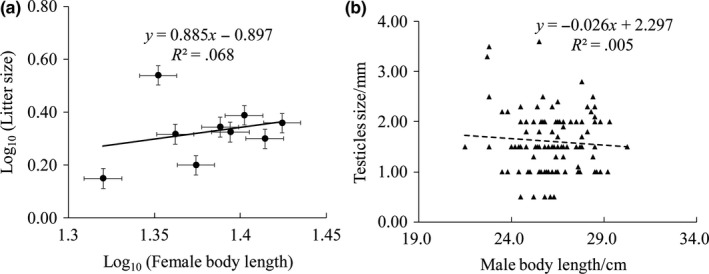
Linear regression between log (litter size) on log (female body size) in females (a) and distribution of testicle size to body size in males (b) plateau zokors

Linear regression of log_10_ (litter size) on log_10_ (female body size) in plateau zokors was nonsignificant (*R* ^2^ = .015, *p *=* *. 409; Figure 6a). Likewise, linear regression of log_10_ (testicular size) on log_10_ (male body size) was nonsignificant (*R* ^2^ = .005, *p *=* *. 434). The frequency of distribution of testicular size revealed that the testicles were larger in medium‐sized males than in lighter or heavier individuals (Figure 6b).

## 4 DISCUSSION

Sexual size dimorphism is one of the most noteworthy peculiarities of nature that is common in animals. In this study, we used measurement data of plateau zokor to investigate the direction of SSD in this species and used the BL of plateau zokors and other seven zokor species to test Rensch's rule. Our results confirmed the occurrence of MBSSD and a significant concordance with Rensch's rule in plateau zokor, but the relationship between the degree of SSD and body size did not follow Rensch's rule in the eight zokors species.

### 4.1 Morphometric analysis of skull and body shapes of plateau zokors

Sexual size dimorphism was detected in the skull and body measurements of plateau zokors. The head of the plateau zokor plays an important role, allowing them to acclimate to the underground lifestyle: It is used to dig and push the loosened soil to the surface during burrowing (Li, Liu, Frelich, & Sun, 2011). Our results suggested that male plateau zokors have heads, especially SL, SBL, ML, ZW, UDL, and DL, larger than those of females. Generally, subterranean rodents possess three mechanisms for burrowing, in which two of them depend uniquely on the head and incisor teeth movements, whereas the third mechanism involves scratch digging using forelimbs (Lin et al., 2010). Incisors are also regularly used for shattering compressed soil particles or cutting the roots of plants when digging burrows (Su, 1992). Both sexes dig extensive burrows for food hoarding, foraging, and protection, and female burrows are deeper (2 m depth) than those of males (1.5 m; Shao et al., 2015). However, the home range of male zokors (1790 ± 720 m^2^) is significantly larger than that of females (260 ± 112 m^2^; Zhang, Fan, & Zhou, 1993); therefore, males are likely to dig more often than females. In addition, during the breeding season, males and females do not live in the same burrow. Males dig longitudinal tunnels with branches intercepting with the female burrows (Zhang, 2007). Indeed, those extra “digging” duties might have resulted in the evolution of MBSSD in skull metrics, that is, length of incisors of the upper and lower jaws for attachment of stronger muscles. This, in turn, might provide remarkable suitability to their underground activities, including locomotion, mate searching, and foraging (Zhao et al., 2016). Perhaps females had undergone similar evolutionary pressures but less extensive than those encountered in males. A parallel explanation could help understand the importance of larger forefoot lengths in male zokors than in females (Echeverría, Biondi, Becerra, & Vassallo, 2016). Measurements of tympanic bulla also showed MBSSD. Zokors have weak vision (Kott, Sumbera, & Nemec, 2010) because of the underground living conditions; signals of predators and competitors are conveyed through low‐frequency acoustic stimuli (Amaya, Areta, Valentinuzzi, & Zufiaurre, 2016; Hegab, Kong, Yang, Mohamaden, & Wei, 2015). Plateau zokors are highly territorial, solitary, and aggressive toward each other, irrespective of sex (Zhang et al., 1993); therefore, males are responsible to guard a larger home range, which might result in the evolution of a more efficient auditory system to receive low‐frequency auditory signals (Squarcia et al., 2007). Nevertheless, the previous assumption does not exclude the fact that the enlargement of tympanic bulla in males might be an outcome of the larger body size.

Sexual size dimorphism is also very clear in terms of BL, BW, and CW. Male zokors have longer bodies and are heavier than females. Notably, the male‐to‐female ratio of average BL was 1.100. This confirms the assumption that the direction of sexual dimorphism in plateau zokors is male‐oriented. MBSSD might be a result of sexual selection, through either intrasexual competition or female choice (preference for larger males; Kelly, Luc, Bussière, & Gwynne, 2008; Himuro & Fujisaki, 2014; Holveck, Gauthier, & Nieberding, 2015; Rohner, Blanckenhorn, & Puniamoorthy, 2016). Further, the “increased‐energy” hypothesis (Shillington & Peterson, 2002) might explain the MBSSD in subterranean zokors endemic to the QTP. This hypothesis was originally explained by Bergmann (1847): In colder climates and at higher altitudes, animals with larger bodies can conserve energy more competently because they own smaller surface‐to‐volume ratio. The activities in males, such as home range keeping, digging and excavation, intrasexual competition, as well as mate and food searching, increase the energy expenditure (Scantlebury, Speakman, & Bennett, 2006). Therefore, a functional evolutionary mechanism by increasing body size is not only beneficial to males to conserve energy expenditure but also enables them to efficiently perform their various daily life tasks. The Bergmannian trends had been also proved in some studies (Meiri et al. 2004, Bidau & Martı 2007); however, our previous assumption may deserve further investigation and confirmation as a more specific study by Medina et al. (2007) on subterranean rodents confirmed that a species of genus *Ctenomys* (*Caviomorpha, Ctenomyidae*) follow the converse to Bergmann's rule.

### 4.2 Development of different morphological traits during aging

A unique perspective regarding the life history confirmed that SSD is proximately facilitated by changes in organism growth and development (Roff, 1992), which in turn are controlled by endocrine, cellular, and physiological mechanisms to obtain maximum benefits for individuals (Nijhout, 2003). SSD might result from the difference in neonatal weight which one sex might be larger at the beginning (Blomquist & Williams, 2013). Alternatively, the variation in body size might result from faster growth rates of one of the sexes, or individuals of one sex might grow for a longer time than the opposite sex (Blanckenhorn et al., 2007). Our results revealed that different age‐groups showed alterations in different morphological traits mainly owing to the growth and maturation of animals that might also reflect the different habits between males and females for the ingestion of diverse food items for heat production in cold climates in addition to other physiological regulators of food intake (Patterson & Abizaid, 2013). The bigger morphological traits in males in group I during the initial stages of development validate the assumption that SSD starts as early as possible, which supports the body for further growth and development as age advances (Le Galliard, Massot, Landys, Meylan, & Clobert, 2006). In groups II, III and IV, SSD was significantly evident in different body measurements. Finally, in the last group (V), UDL and FFL traits lost their male‐biased SSD (Table S1). The FFL may serve males to dig larger home ranges for breeding purpose (Lin et al., 2010), a function that will inevitably diminished in older ages which may indicate reproductive aging in males. Moreover, the nonsignificant difference in UDL between the two sexes might indicate that the feeding habits and nutritional requirements for both sexes at an older age are similar (Xie et al., 2014).

The *t* test analysis showed that tail length varies significantly between sexes: Males have longer tails than females. The tail in some subterranean rodent species serves multiple purposes, including dirt removal (Bathergids), orientation during excavation (*Geomys bursarius*), and dumping heat (Geomyids), and in *Ctenomys* to support body in concert with hind or forefeet (Stein, 2000). Because males are more active than females, we assume that tails might play a vital role to direct animal movements in the underground environment. Body weights significantly increased with age, which also supports the notion that SSD might result from faster growth rates in one of the sexes. The similarities and differences of sex traits through age can reveal the ecological functions and reasons underlying the development of SSD in plateau zokors.

### 4.3 The relationships between morphological indicators and CW, BW, and BL

Our results showed that the linear regression of different morphometric traits correspondingly increased with the increment in CW, BW, and BL in both sexes, with a higher degree of correlation found in males than in females. These findings suggest that the growth rate of males is faster than that of females. This could be the reason for the male‐biased SSD in plateau zokors (John‐Alder, Cox, & Taylor, 2007). Remarkably, the most consistently dimorphic traits were the length of the skull and the lower incisor length (Figures 2, 3, and 4) that were significantly higher in males and might be related to the excavation of soil for constructing and maintaining burrows, and keeping the bigger home range (Becerra, Casinos, & Vassallo, 2013).

Part of the rodent control and prevention strategies depends on a comprehensive understanding of the reproductive traits of males and females. We assessed the morphological characteristics of testicles and litter sizes in relation to body sizes of both sexes. Strikingly, the body size of females did not show any linear relationship with litter sizes (Figure 6a). Our results did not corroborate the assumption of the fecundity selection hypothesis (Pincheira‐Donoso & Hunt, 2017) that larger females can reproduce more efficiently than smaller ones, suggesting that other underlying factors might regulate the evolution of body size in plateau zokors. Moreover, males followed the same pattern, and the size of testicles was not linearly correlated with the male body size (See results section 3.4). The frequency of higher testicular size was greater in medium‐sized individuals (Figure 6b). Although the size of testes in mammals and other organisms might be considered as an indicator of the reproductive strategies and can be utilized in the context of evolutionary biology and sexual selection theory, similar studies on tuco‐tucos (*Ctenomys*) revealed that small males might possess bigger testicular size. A possible explanation was that, unlike larger males, smaller males would invest more in testes growth and thus ejaculate quality, which would compensate for their apparently lower chances of accessing females because of their smaller body size (Schulte‐Hostedde & Millar, 2004).

A standard approach, which we followed to test Rensch's rule, is to determine whether a bivariate plot of log_10_ (female size) versus log_10_ (male size) has a slope significantly different from 1.0. We graphically plotted log (female size/*x*‐axis) on log (male size/*y*‐axis) in plateau zokors (Fairbairn, 1997), and the slope was statistically found to be higher than one (Figure 5a; Wu et al., 2014). This was found not only in plateau zokors but also data obtained from the different zokor species yielded similar results (Figure 5b). However, an alternative method was used taken into consideration the phylogenetic relationship between different zokors species. βRMA (slope of the RMA regression) is not significantly >1.0, which in turn would signal nonconcordance with Rensch's rule between different zokor species. Our findings are consistent with the assumption proposed by Martínez and Bidau (2016) “It could be inferred that the subterranean lifestyle does not impose a single scaling pattern of SSD at the intraspecific level but that conformity to Rensch's rule or not, is a species‐specific characteristic.” According to our findings and those from Martínez and Bidau (2016), Plateau zokor (*Eospalax baileyi*) conforms to Rensch's rule while other seven zokors species, although they have the same underground lifestyle, may not uniformly follow Rensch's rule.

In conclusion, our results emphasize the intricacy of the nature and evolution of SSD in plateau zokors. We found that the direction of SSD in plateau zokors was male‐biased and the variation pattern in SSD was consistent with Rensch's rule in plateau zokors but not in the other seven zokor species. Several factors contribute to the development of MBSSD in plateau zokors, which include geographical, ecological, and behavioral aspects. These data suggest that the skull morphometric variation of zokors evolves under natural selection for improving the ability to dig and to conduct other activities underground. Moreover, significant differences in the functional traits between the two sexes suggest that task‐dependent evolutionary changes in males play a role in the covariation of head shape and body size between the sexes. Previous studies have suggested the various motives for allometry for SSD, but most available evidence from our findings did not support the view that only sexual selection on male body size was the main contributing factor to the existence of SSD, but several factors may contribute to this phenomenon.

## AUTHOR CONTRIBUTIONS

JS carried out the experiment. JS, ImH, WJ, and ZN wrote the manuscript. ZN supervised the project.
