# Supplementary material for: Function‐related Drivers of Skull Morphometric Variation and Sexual Size Dimorphism in a Subterranean Rodent, Plateau Zokor (Eospalax baileyi)
Source: Ecol Evol. 2018 Apr 15;8(9):4631–43. doi: 10.1002/ece3.3986 (PMC5938458; doi:10.1002/ece3.3986)
Supplement: Supplementary file 1 [file ECE3-8-4631-s001.docx]

| **Item** | **GROUP I** | | ***p* value** | **GROUP II** | | ***p* value** | **GROUP III** | | ***p* value** | **GROUP IV** | | ***p* value** | **GROUP V** | | ***p* value** | ***P* value (between groups)** |
| --- | --- | --- | --- | --- | --- | --- | --- | --- | --- | --- | --- | --- | --- | --- | --- | --- |
|  | **Male** | **Female** |  | **Male** | **Female** |  | **Male** | **Female** |  | **Male** | **Female** |  | **Male** | **Female** |  |  |
| **BL/cm** | 24.76  ±  0.31 | 23.23  ±  0.25 | *P<0.001* | 26.06  ±  0.19 | 23.73  ±  0.11 | *P<0.001* | 26.70  ±  0.18 | 24.80  ±  0.12 | *P<0.001* | 27.49  ±  0.37 | 24.36  ±  0.37 | *P<0.001* | 26.53  ±  0.44 | 23.27  ±  0.43 | *p=*0.003 | *P<0.001* |
| **SL/mm** | 44.21  ±  0.46 | 42.29  ±  0.49 | *p=0.008* | 45.40  ±  0.23 | 42.82  ±  0.14 | *P<0.001* | 46.46  ±  0.24 | 43.69  ±  0.51 | *P<0.001* | 47.71  ±  0.30 | 43.87  ±  0.54 | *P<0.001* | 48.79  ±  0.64 | 44.03  ±  0.72 | *p=*0.003 | *P<0.001* |
| **SBL/mm** | 42.43  ±  0.50 | 40.54  ±  0.51 | *p=0.014* | 44.16  ±  0.22 | 41.33  ±  0.16 | *P<0.001* | 45.34  ±  0.26 | 42.64  ±  0.26 | *P<0.001* | 46.39  ±  0.36 | 42.47  ±  0.50 | *P<0.001* | 47.76  ±  0.70 | 42.43  ±  0.66 | *p=*0.002 | *P<0.001* |
| **DL/mm** | 9.73  ±  0.08 | 9.61  ±  0.11 | *p=0.372*  ***NS*** | 9.91  ±  0.04 | 9.67  ±  0.03 | *P<0.001* | 10.14  ±  0.05 | 9.88  ±  0.04 | *P<0.001* | 10.30  ±  0.07 | 9.89  ±  0.13 | *P<0.001* | 10.33  ±  0.13 | 10.00  ±  0.40 | *p=*0.32  ***NS*** | *P<0.001* |
| **ML/mm** | 14.71  ±  0.15 | 13.97  ±  0.20 | *p=0.006* | 15.45  ±  0.10 | 14.44  ±  0.07 | *P<0.001* | 15.85  ±  0.10 | 15.04  ±  0.09 | *P<0.001* | 16.08  ±  0.13 | 14.91  ±  0.19 | *P<0.001* | 17.07  ±  0.26 | 14.53  ±  0.22 | *P<0.00* | *P<0.001* |
| **TBL/mm** | 9.27  ±  0.09 | 8.89  ±  0.11 | *p=0.013* | 9.49  ±  0.05 | 9.13  ±  0.04 | *P<0.001* | 9.58  ±  0.06 | 9.36  ±  0.04 | *p=0.004* | 9.61  ±  0.07 | 9.40  ±  0.17 | *p=0*.20  ***NS*** | 9.92  ±  0.09 | 9.43  ±  0.18 | *P<0.00* | *P<0.001* |
| **TBW/mm** | 7.19  ±  0.08 | 7.02  ±  0.08 | *p=0.164*  ***NS*** | 7.44  ±  0.05 | 7.16  ±  0.04 | *P<0.001* | 7.50  ±  0.05 | 7.32  ±  0.05 | *p=0.011* | 7.56  ±  0.07 | 7.18  ±  0.13 | *p=0*.02 | 7.40  ±  0.08 | 7.03  ±  0.09 | *p=*0.043 | *p=0.02* |
| **OB/mm** | 8.14  ±  0.04 | 8.08  ±  0.09 | *p=0.536*  ***NS*** | 8.31  ±  0.05 | 8.19  ±  0.04 | *p=0.079*  ***NS*** | 8.21  ±  0.05 | 8.11  ±  0.06 | *p=0.196*  ***NS*** | 8.38  ±  0.05 | 8.00  ±  0.08 | *P<0.001* | 8.30  ±  0.13 | 7.93  ±  0.18 | *p=*0.18  ***NS*** | *p=0.26*  ***NS*** |
| **NL/mm** | 15.99  ±  0.25 | 14.94  ±  0.27 | *p=0.007* | 16.50  ±  0.22 | 15.33  ±  0.09 | *P<0.001* | 17.21  ±  0.16 | 16.07  ±  0.11 | *P<0.001* | 17.74  ±  0.20 | 16.19  ±  0.26 | *p*<0.00 | 18.06  ±  0.40 | 16.00  ±  0.15 | *p=*0.02 | *P<0.001* |
| **ZW/mm** | 30.48  ±  0.40 | 29.23  ±  0.78 | *p=0.148*  ***NS*** | 31.65  ±  0.25 | 29.09  ±  0.19 | *P<0.001* | 33.19  ±  0.28 | 30.54  ±  0.20 | *P<0.001* | 34.38  ±  0.42 | 30.30  ±  0.45 | *P<0.001* | 36.47  ±  1.23 | 29.67  ±  1.01 | *p=*0.009 | *P<0.001* |
| **OW/mm** | 27.85  ±  0.39 | 26.45  ±  0.50 | *p=0.038* | 29.33  ±  0.21 | 27.04  ±  0.14 | *P<0.001* | 30.48  ±  0.22 | 28.15  ±  0.17 | *P<0.001* | 31.59  ±  0.33 | 28.00  ±  0.32 | *P<0.001* | 32.35  ±  0.56 | 27.60  ±  0.67 | *p=*0.001 | *P<0.001* |
| **MPL/mm** | 18.89  ±  0.20 | 18.14  ±  0.21 | *p=0.013* | 19.41  ±  0.20 | 18.62  ±  0.08 | *P<0.001* | 20.31  ±  0.16 | 19.23  ±  0.09 | *P<0.001* | 20.62  ±  0.16 | 19.20  ±  0.14 | *P<0.001* | 21.50  ±  0.29 | 18.70  ±  0.31 | *P<0.001* | *P<0.001* |
| **UDL/mm** | 9.99  ±  0.08 | 9.77  ±  0.10 | *p=0.082*  ***NS*** | 10.14  ±  0.05 | 9.85  ±  0.03 | *P<0.001* | 10.34  ±  0.05 | 10.05  ±  0.04 | *P<0.001* | 10.43  ±  0.07 | 10.20  ±  0.18 | *p=0*.187  ***NS*** | 10.51  ±  0.15 | 10.17  ±  0.23 | *p=*0.30  ***NS*** | *P<0.001* |
| **JL/mm** | 28.23  ±  0.27 | 27.11  ±  0.36 | *p=0.019* | 29.28  ±  0.15 | 27.78  ±  0.11 | *P<0.001* | 30.01  ±  0.17 | 28.74  ±  0.10 | *P<0.001* | 30.67  ±  0.26 | 28.83  ±  0.16 | *p=0*.001 | 31.63  ±  0.52 | 28.53  ±  0.41 | *p=*0.01 | *P<0.001* |
| **TL/mm** | 4.67  ±  0.10 | 4.23  ±  0.12 | *p=0.007* | 4.89  ±  0.07 | 4.29  ±  0.05 | *P<0.001* | 4.83  ±  0.07 | 4.47  ±  0.07 | *P<0.001* | 5.03  ±  0.14 | 4.42  ±  0.23 | *p=*0.04 | 5.13  ±  0.11 | 4.30  ±  0.38 | *p=*0.01 | *p=*0.30  ***NS*** |
| **FFL/mm** | 3.04  ±  0.02 | 3.01  ±  0.02 | *p=0.193*  ***NS*** | 3.11  ±  0.02 | 3.06  ±  0.01 | *p=0.024* | 3.19  ±  0.02 | 3.10  ±  0.02 | *P<0.001* | 3.28  ±  0.04 | 3.02  ±  0.07 | *p=0*.003 | 3.26  ±  0.04 | 2.83  ±  0.17 | *p=*0.12  ***NS*** | *p<0.01* |
| **HFL/mm** | 3.35  ±  0.05 | 3.20  ±  0.03 | *p=0.011* | 3.52  ±  0.03 | 3.30  ±  0.01 | *P<0.001* | 3.61  ±  0.03 | 3.45  ±  0.02 | *P<0.001* | 3.73  ±  0.05 | 3.39  ±  0.05 | *p=0*.001 | 3.89  ±  0.04 | 3.30  ±  0.10 | *P<0.00* | *P<0.001* |
| **CW (g)** | 201.24  ±  11.62 | 157.29  ±  4.38 | *p=0.001* | 238.99  ±  4.01 | 179.77  ±  2.04 | *P<0.001* | 303.41  ±  3.05 | 219.01  ±  2.99 | *P<0.001* | 369.64  ±  5.26 | 228.80  ±  12.02 | *P<0.001* | 403.11  ±  36.35 | 249.30  ±  36.26 | *p=*0.02 | *P<0.001* |
| **BW (g)** | 247.11  ±  5.06 | 203.07  ±  3.32 | *p<0.00* | 323.42  ±  3.35 | 253.15  ±  1.69 | *P<0.001* | 398.83  ±  3.02 | 303.75  ±  2.14 | *P<0.001* | 478.85  ±  3.81 | 363.56  ±  6.45 | *P<0.001* | 554.57  ±  5.69 | 449.30  ±  2.95 | *P<0.00* | *P<0.001* |
